# X-ray irradiation selectively kills thymocytes of different stages and impairs the maturation of donor-derived CD4^+^CD8^+^ thymocytes in recipient thymus

**DOI:** 10.7555/JBR.26.20120003

**Published:** 2012-06-08

**Authors:** Jinbo Li, Hongquan Cai, Jianliang Jin, Qian Wang, Dengshun Miao

**Affiliations:** a The Research Center for Bone and Stem Cells, Department of Human Anatomy, Nanjing Medical University, Nanjing, Jiangsu 210029, China;; b Department of Preventive Medicine, School of Public Health, Nanjing Medical University, Nanjing, Jiangsu 210029, China.

**Keywords:** thymus, radiation, CD4^+^CD8^+^ thymocytes, sensitivity, donor cells

## Abstract

The aim of the present study was to determine whether the sensitivity of thymocytes to X-ray radiation depends on their proliferative states and whether radiation impairs the maturation of donor-derived thymocytes in recipient thymus. We assigned 8-week-old C57BL/6J mice into three treatment groups: 1) untreated; 2) X-ray radiation; 3) X-ray radiation plus bone marrow transplantation with donor bone marrow cells from transgenic mice expressing enhanced green fluorescent protein (GFP) on a universal promoter. After 4 weeks, the size of the thymus, the number and proliferation of thymocytes and ratios of different stage thymocytes were analyzed by immunohistochemistry and flow cytometry. The results showed that: 1) CD4^+^CD8^+^ thymocytes were more sensitive to X-ray radiation-induced cell death than other thymocytes; 2) the proliferative capacity of CD4^+^CD8^+^ thymocytes was higher than that of other thymocytes; 3) the size of the thymus, the number of thymocytes and ratios of thymocytes of different stages in irradiated mice recovered to the normal level of untreated mice by bone marrow transplantation; 4) the ratio of GFP-positive CD4^+^CD8^+^ thymocytes increased significantly, whereas the ratio of GFP-positive CD4^+^ or CD8^+^ thymocytes decreased significantly. These results indicate that the degree of sensitivity of thymocytes to X-ray radiation depends on their proliferative states and radiation impairs the maturation of donor-derived CD4^+^CD8^+^ thymocytes in recipient thymus.

## INTRODUCTION

T-cell development in the thymus is a complex process. CD4^–^ CD8^–^ double-negative (DN) thymocytes develop from early thymic progenitors located in the subcapsular zone (SCZ), and then advance to CD4^+^CD8^+^ double-positive (DP) cells as they meander through the cortex. DP thymocytes in the cortex undergo a critical developmental checkpoint called positive selection and a small fraction of DP thymocytes successfully complete positive selection, travel to the medulla and become CD4^+^ or CD8^+^ single-positive (SP) thymocytes. In the medulla, negative selection is required for eliminating self-reactive thymocytes. Mature thymocytes in the medulla egress from the thymus to the blood stream and then migrate to the peripheral lymphoid organs[1],[2].

It is known that ionizing radiation causes organ atrophy and inhibition of the immune system and the underlying mechanism involves radiation-induced apoptosis in immunocytes[3]. Furthermore, in the thymus, non-immune structural cells such as thymic epithelial cells, which are recognized as one of the important components of the microenvironment for T-cell development, are also damaged.

Immature DP thymocytes, a major component of the thymus, are intrinsically sensitive to p53-dependent or independent apoptosis induced by radiation[4]–[7]. Previous studies mainly focused on radiation-induced apoptosis and its underlying mechanism. However, whether DN and SP thymocytes could completely escape from radiation-induced cell death and what contributes to the different cell fates of DN, DP and SP thymocytes remains to be elucidated.

Once lymphoid precursors enter the thymus from the blood stream, they come into contact with thymic epithelial cells that guide their maturation into functionally competent T cells[8]. Some studies have proven that radiation could induce microenvironment damage, which plays an active role in carcinogenesis[9]. Total body radiation has been widely used clinically to eliminate malignant cells or to inhibit immune response for bone marrow transplantation (BMT). Therefore, whether donor-derived precursors could develop normally in the thymus of irradiated recipient is drawing increasing attention.

Here, we designed this animal experiment to determine whether the sensitivity of thymocytes to X-ray radiation (XR) depends on their proliferative states and whether radiation impairs the maturation of donor-derived thymocytes in recipient thymus.

## MATERIALS AND METHODS

### Mice

C57BL/6J (WT) mice (≤8 weeks) and transgenic mice expressing enhanced GFP were purchased from the Jackson Laboratory (Bar Harbor, Maine, USA) and Riken BioResource Center (Tsukuba, Ibaraki, Japan), respectively. The mice were maintained in the Experimental Animal Center of Nanjing Medical University. All experimental procedures were carried out in accordance with the United States National Institutes of Health Guidelines for Care and Use of Laboratory Animals. C57BL/6J (WT) mice were assigned into three treatment groups: 1) untreated control; 2) XR; 3) XR+BMT (transplanted with bone marrow cells from transgenic mice expressing EGFP).

### XR and BMT

The genotypes of donor and recipient mice used in this study are EGFP transgenic mice and C57BL/6J wildtype mice, respectively. At 8 weeks of age, recipient mice first received 9.5 Gy single-dose total body irradiation by a Varian 600CD linear accelerator. Within 24 h, 5×10^6^ donor cells suspended in 100 µL α-minimal essential medium (α-MEM, Invitrogen, Carlsbad, CA, USA) were administered via the tail vein as previously described[10]. Four weeks after transplantation, recipient mice were euthanized and the respective thymus tissues were prepared for further analysis.

### BrdU incorporation assay

One dose of 3 mg BrdU (Sigma, St. Louis, MO, USA) was intraperitoneally (i.p.) injected into mice. Bone marrow (BM) samples were collected 3 d later for further analysis.

### Flow cytometry

Cell surface labeling: Single-cell suspensions of thymocytes stained with FITC-CD4, PE-CD8α or APC-CD8α (eBioscience, San Diego, CA, USA) were analyzed by flow cytometry. All labeling was performed in a sodium potassium buffer containing glucose and 1% BSA for 30 min at 4°C. After centrifugation, the cells were washed with phosphate buffered saline (PBS), fixed with 2% formalin and subsequently analyzed on a FACSCalibur flow cytometer (BD Biosciences, Franklin Lakes, NJ, USA).

BrdU staining: Single-cell suspension of thymocytes was stained using the above fluorescence labeling antibodies targeting CD4 and CD8, and then fixed with 95% alcohol for 10 min in 4°C. The fixed cells were washed and incubated with a mouse anti-BrdU antibody (Millipore, Billerica, MA, USA) for 30 min. The cells were then washed again and incubated with an APC-conjugated goat anti-mouse IgG (Biolegend, San Diego, CA, USA) following the manufacturer's instructions.

### Cell cycle analysis

Totally, 1×10^6^ mouse thymus cells were stained using the cocktail of the above fluorescence labeling antibodies targeting CD4 and CD8 and fixed by 70% ethanol for 1 h at -20°C. After washing with PBS, the cells were stained with 300 µL propidium iodide (PI) staining solution (BD Biosciences) for 30 min on ice. Finally, the sample was kept in the dark for analysis. All data of flow cytometry were analyzed with the FlowJo Version 7.6.1 software (TreeStar Inc., Ashland, OR, USA).

### Polymerase chain reaction (PCR)

DNA was extracted from the thymus and amplified by PCR. PCR was performed with the following protocol: 95°C for 5 min×1 cycle; 94°C for 30 sec, 52°C for 45 s, 72°C for 1 min×35 cycles; 72°C for 2 min×1 cycle; stored at 4°C. The following primers were used: EGFP with 5′-GCC ACA AGT TCA GCG TGT CCG-3′ (forward primer) and 5′-GTT GGG GTC TTT GCT CAG GGC G-3′ (reverse primer). Agarose gel electrophoresis was performed to solve the PCR products of EGFP (565 bp).

### Immunohistochemistry

Immunohistochemical staining for Ki67 and BrdU was performed using an affinity-purified rabbit anti-mouse Ki67 antibody (Abcam, Cambridge, UK) and a mouse anti-BrdU antibody (Millipore), respectively, as described previously[11]. Briefly, dewaxed and rehydrated paraffin-embedded sections were incubated with hydrogen peroxide in methanol (1:10) to block endogenous peroxidase activity and then washed with Tris-buffered saline (TBS, pH 7.6). The sections were then incubated with the primary antibody overnight at room temperature. After rinsing with TBS for 15 min, the sections were incubated with the secondary antibody (biotinylated goat anti-rabbit IgG or biotinylated goat anti-mouse IgG; Sigma, Louis, MO, USA). The sections were then washed and incubated with the Vectastain Elite ABC reagent (Vector Laboratories, Burlington, Ontario, Canada) for 45 min before addition of 2.5 mg/mL 3,3-diaminobenzidine (DAB). Finally, the stained sections were counterstained with hematoxylin and eosin (H&E) staining[12]. Images were acquired with a Leica microscope (Leica DM4000B, Solms, German) equipped with Leica software.

### Statistical analysis

Experiments were independently performed for at least 3 times. Student's *t*-test and one-way ANOVA with Fisher's least significant difference (LSD) post hoc test were used to analyze the statistical significance among thymic parameters. A Pearson correlation analysis was performed to investigate the relationship between the percentages of thymocytes maintained at G_2_/M phase before radiation and the percentages of radiation-induced cell death 4 weeks after radiation in different-stage populations. Statistical analysis was performed using SPSS 16.0 (SPSS Institute, Chicago, IL, USA) and the data in tables are expressed as mean±SD. The data in figures were analyzed by GraphPad Prism 5 software (GraphPad Software, San Diego, CA, USA) and were expressed as mean±SD. *P* < 0.05 was considered statistically significant.

## RESULTS

### Thymocytes at different stages exhibit different radiosensitivities

**Fig. 1:**
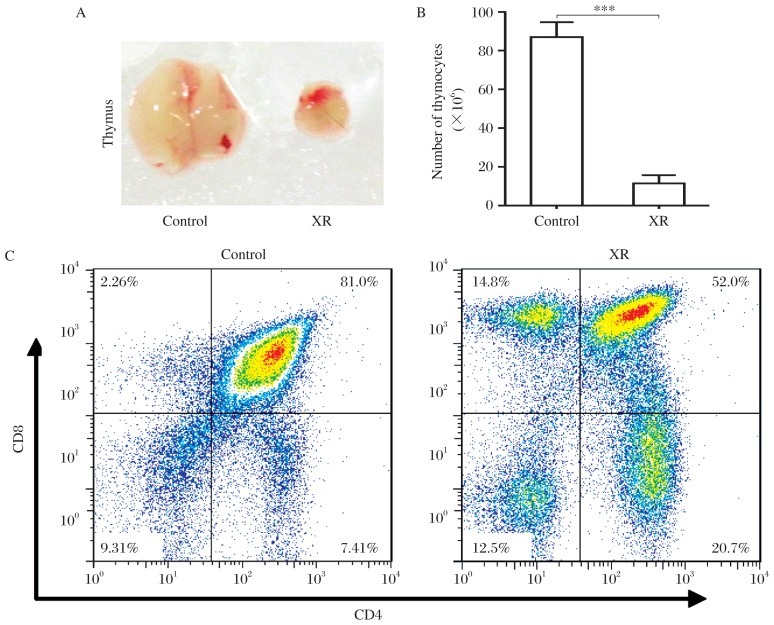
Different sensitivities to X-ray-induced cell death in thymocytes at different stages. A: Representative gross graph of the thymus from 12-week-old non-irradiated mice (untreated control) and X-ray irradiated mice (XR) with a dose of 9.5 Gy; B: Bar graph shows the total number of thymocytes in each group (mean±SD; ***P < 0.001; *n* = 5 each). C: Representative figures of flow cytometric analysis of CD4 and CD8 expression in thymocytes in the untreated and irradiated mice 4 weeks after irradiation.

To determine the change in the thymus after radiation, we first evaluated the size and cellularity of the thymus in untreated and irradiated mice. The results showed that, at 4 weeks after irradiation with 9.5 Gy dose, the size of the thymus and the number of thymocytes decreased significantly in the irradiated mice compared with untreated mice (***Fig. 1A*** and ***1B***). Flow cytometric analysis revealed that the ratio of DP thymocytes decreased significantly, whereas that of CD4^–^CD8^–^ (DN), CD4^+^CD8^–^ (CD4-SP) and CD4^–^CD8^+^ (CD8-SP) thymocytes increased significantly in the irradiated mice compared with untreated mice (***Fig. 1C***). However, the numbers of DP, DN, CD4-SP and CD8-SP thymocytes decreased significantly in the irradiated mice to 7.9%, 23.6%, 40.2% and 80.3% of untreated mice, respectively (***Table 1***). These results revealed that all different stage thymocytes could be affected when exposed to irradiation, and DP thymocytes were the most severely affected while other thymocytes were less affected.

**Table 1:** Effects of radiation on thymocytes of different stages

Group	Constituent ratio (%)		Cellularity (×10^6^)		Cell death (%) (1-XR/Untreated) ×100%
	Untreated	XR	Untreated	XR	
CD4^–^CD8^–^	08.21±2.08	14.17±1.88*	07.07±1.55	1.67±0.85**	76.38
CD4^+^CD8^+^	82.63±2.10	49.87±1.91***	71.95±7.97	5.70±2.08***	92.08
CD4^+^CD8^–^	06.65±0.69	20.53±1.46***	05.75±0.07	2.31±0.69**	59.83
CD4^–^CD8^+^	002.5±0.50	015.4±0.60***	02.19±0.59	1.76±0.66	19.63

*P < 0.05, **P < 0.01, ***P < 0.001 compared with the untreated group. XR: irradiation.

(mean±SD, *n* = 5)

**Fig. 2:**
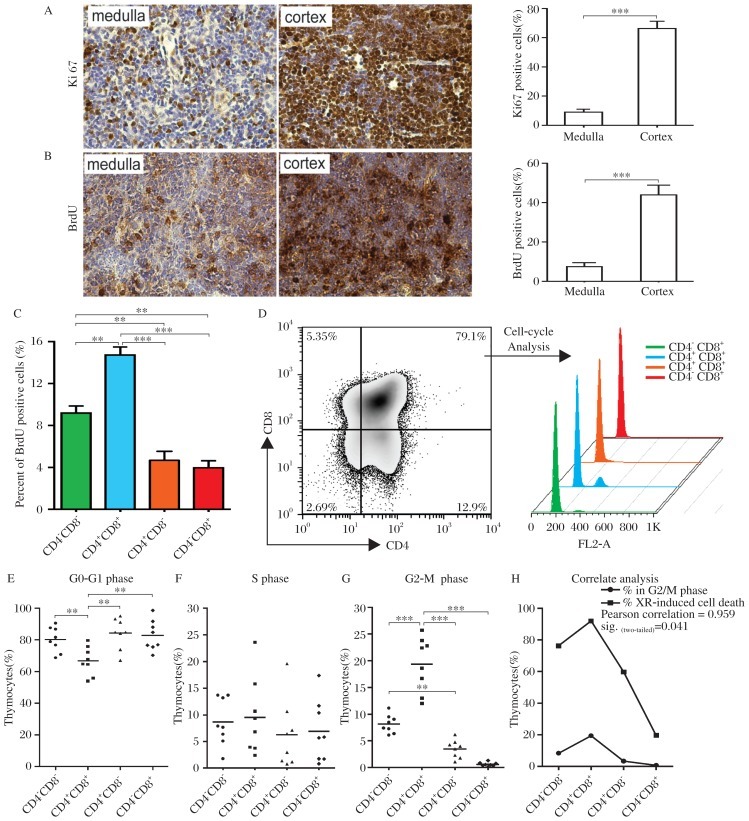
The degree of radiosensitivity of thymocytes depends on their proliferative states. A: Representative tissue sections of the thymus stained immunohistochemically for Ki67. The right panel is the percentage of Ki67 positive cells in the cortex and the medulla of the thymus from 8-week-old C57BL/6J (WT) mice (mean±SD; ***P < 0.001; *n* = 3 each); B: Thymocyte proliferation was tested by BrdU incorporation assay for 3 days and by immunohistochemistry staining for BrdU. The right panel is the percentage of BrdU positive cells in the cortex and the medulla of the thymus (mean±SD; ***P < 0.001; *n* = 3 each). C: The percentage of BrdU positive cells in thymocytes detected by flow cytometry at different stages is presented (mean±SD; **P < 0.01, ***P < 0.001; *n* = 6 each). D: Thymocytes were triple-stained with CD4, CD8 and propidium iodide (PI) to reveal the cell-cycle status of thymocytes of different stages. Representative cell-cycle states of thymocytes of different stages from one thymus are demonstrated on the right panel. The percentages of G_0_/G_1_ phase (E), S phase (F) and G_2_/M phase (G) in thymocytes of different stages from 8 independent mice were statistically analyzed (mean±SD; *P < 0.05, **P < 0.01, ***P < 0.001; *n* = 8 each). H: Correlation analysis was performed between the percentages of G_2_/M phase thymocytes and the percentages of radiation-induced cell death in each thymocytes of different stages (Pearson correlation = 0.959*).

### Radiosensitivity of thymocytes at different stage depends on their proliferative states

**Fig. 3:**
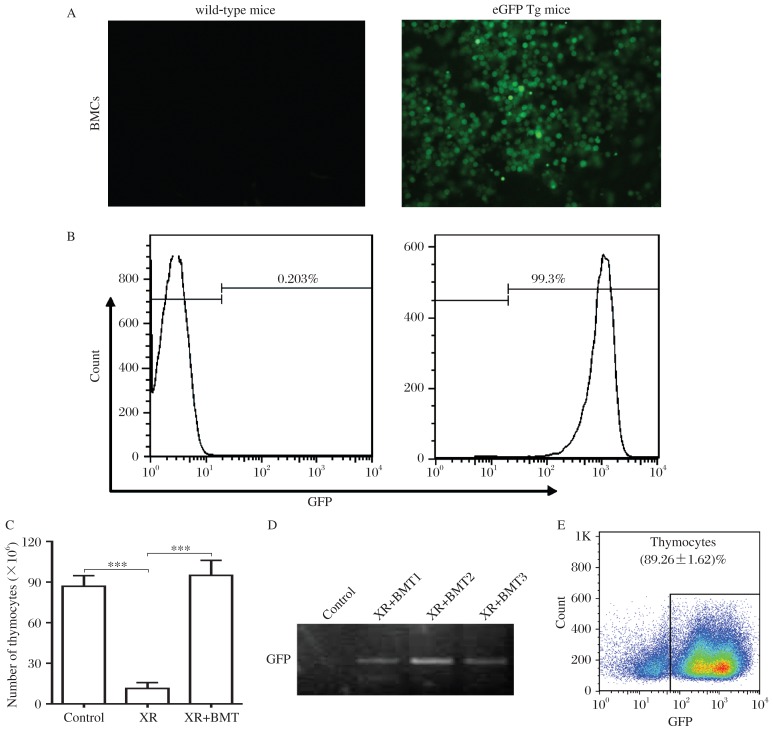
Donor cells contributed to thymus regeneration in transplant recipients. A: Representative fluorescence micrographs for GFP expression (original magnification×400) in BM cells (BMCs) from C57BL/6J wildtype (WT) mice and EGFP transgenic (EGFP Tg) mice. B: Flow cytometric analysis of GFP expression in BMCs from WT mice and EGFP Tg mice. C: The bar graph showed the total numbers of thymocytes from control, XR and the irradiated mice with BMT (mean±SD; ***P < 0.001, *n* = 5 each). D: GFP gene expression in the thymuses from the control and XR+BMT mice. E: Flow cytometric analysis for the percentage of GFP-positive thymocytes in the thymus from the irradiated mice with BMT at 4 weeks after transplantation. Data are expressed as mean±SD (*n* = 5).

Previous studies have reported that DNA double-strand breaks constitute the most dangerous type of DNA damage induced by ionizing radiation, and genomic instability induced by perturbed recombination in cancer cells make them sensitive to ionizing irradiation[13]. Therefore, we hypothesized that the degree of radiosensitivity of thymocytes at different stages depends on their proliferative states. To test this hypothesis, the proliferation of thymocytes from C57BL/6J mice (≤8 week) before radiation was detected. The results of Ki67 immunohistochemistry revealed that the percentage of Ki67 positive cells was higher in the cortex than that in the medulla (***Fig. 2A***). To further confirm this result, we performed a 3-d BrdU incorporation assay to evaluate the proliferation of thymocytes. The results of BrdU incorporation assay also confirmed that the percentage of BrdU-positive cells was higher in the cortex than that in the medulla (***Fig. 2B***). In view of the fact that immature DN and DP thymocytes are localized principally in the SCZ and cortical regions, respectively, and mature SP thymocytes reside within the inner thymic medulla[8], we further analyzed the percentage of BrdU-positive thymocytes in different developmental stages using flow cytometry. The results showed that the percentages of BrdU-positive thymocytes in DN, DP, CD4-SP and CD8-SP populations were 9.2%, 14.7%, 4.7% and 4.0%, respectively. The percentages of BrdU-positive thymocytes in DN and DP populations were higher than those in CD4-SP and CD8-SP populations (***Fig. 2C***). When cell cycle was analyzed, an obvious G2/M peak was detected in DP thymocytes, but not in the other 3 populations (***Fig. 2D***). Statistical analysis revealed that 1) the percentage of thymocytes at G0/G1 phase in DP population was significantly lower than that in DN, CD4-SP and CD8-SP thymocytes (***Fig. 2E***); 2) the percentage of thymocytes at S phase was not significantly altered in DN, DP, CD4-SP and CD8-SP populations (***Fig. 2F***); 3) the percentage of thymocytes at G_2_/M phase in DP population was significantly higher than that in DN, CD4-SP and CD8-SP populations; 4) the percentage of thymocytes at G_2_/M phase in CD4-SP population was significantly higher than that in DN and CD8-SP populations (***Fig. 2G***). In addition, we performed a correlation analysis to investigate the relationship between the percentages of thymocytes maintained at G_2_/M phase before radiation and the percentages of radiation-induced cell death 4 weeks after radiation in different-stage populations. As shown in ***Fig. 2H***, there was a significant correlation between the two variables, suggesting that the degree of radiosensitivity of thymocytes depends on their proliferative states.

### Donor cells contribute to thymus regeneration in transplant recipients

To assess the role of BM cell transplantation in thymus regeneration in irradiated recipients, 5×10^6^ BM cells harvested from EGFP transgenic mice were transplanted into the irradiated mice. After 4 weeks, the size of the thymus, the number of thymocytes and the percentage of donor-derived GFP-positive thymocytes were analyzed. Fluorescence microscopy and flow cytometry for GFP expression revealed that 99.3% of donor BM cells were GFP-positive (***Fig. 3A*** and ***3B***). At 4 weeks after the transplantation, the size of the thymus and the number of thymocytes in the irradiated mice with BMT recovered to the normal level of untreated mice (***Fig. 3C***). Furthermore, the presence of GFP DNA was confirmed by PCR in thymocytes harvested from recipient mice (***Fig. 3D***) and flow cytometry demonstrated that 89.26±1.62% thymocytes were GFP positive in recipient mice (***Fig. 3E***). Taken together, these results demonstrated that donor BM cells contributed to thymus regeneration in transplant recipients.

### Radiation impairs the maturation of donor cells in recipient thymus

**Fig. 4:**
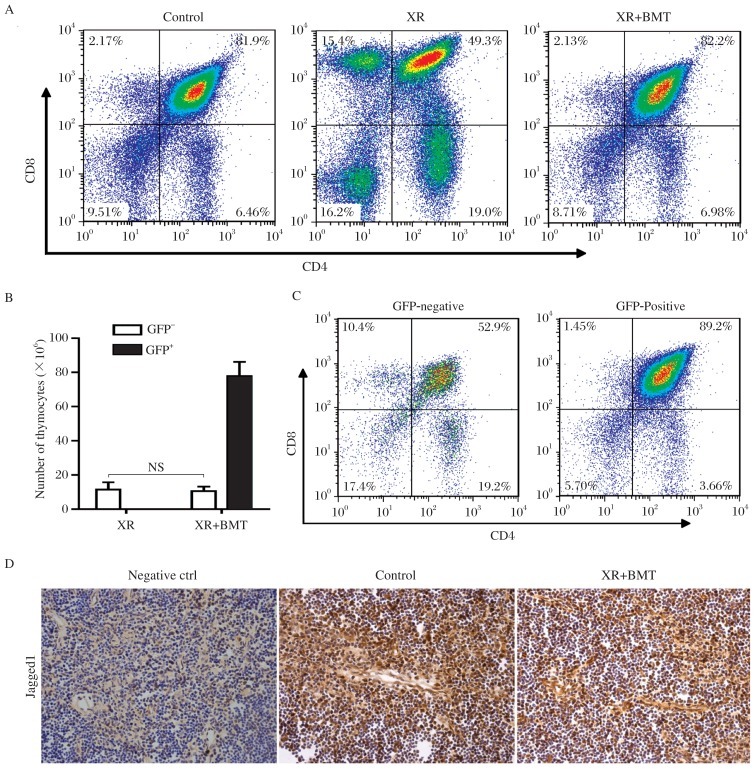
Radiation impairs the maturation of donor cells in recipient thymus. A: Representative flow cytometric analyses for the CD4/CD8 double-stained thymocytes from mice at 4 weeks after transplantation. B: The numbers of GFP-negative or GFP-positive thymocytes in the irradiated mice and the irradiated mice with bone marrow transplantation (BMT), respectively (mean±SD; *n* = 5 each; NS: not significant). C: Representative flow cytometric analyses for the CD4/CD8 double-stained thymocytes in GFP-negative thymocytes and GFP-positive thymocytes from the irradiated mice with BMT. D: Representative tissue sections of thymus stained immunohistochemically for Jagged1 expression in thymic cortical epithelia in the untreated mice and the irradiated mice with BMT.

To assess the effect of BM cell transplantation on the reconstruction of thymocytes of different stages in irradiated recipients, DN, DP, CD4-SP and CD8-SP thymocytes were compared between the irradiated mice with BMTand control mice or XR mice. The results showed that the ratios of DN, DP, CD4-SP and CD8-SP thymocytes recovered to normal level in the irradiated mice with BMT (***Fig. 4A***, ***Table 2***). Compared with XR mice, the ratio of DP thymocytes significantly increased, whereas the ratios of DN and SP thymocytes significantly decreased in the irradiated mice with BMT (***Fig. 4A***, ***Table 2***). To further assess the contribution of donor BM cells in the reconstruction of thymocytes of different stages in irradiated recipients, total thymocytes and thymocytes of different stages derived from recipient and donor were identified using flow cytometric analysis. The results showed that the number of GFP-negative thymocytes from the irradiated mice with BMT was comparable with that in the irradiated mice (***Fig. 4B***). The ratios of DN, DP, CD4-SP and CD8-SP thymocytes in GFP-negative population in the irradiated mice with BMT were comparable with those in total thymocytes from the irradiated mice (***Fig. 4C***, left panel). These results confirmed that the reconstruction of irradiated thymus depended on donor BM cells. In GFP-positive population, the ratio of DN thymocytes did not decrease, whereas the ratio of DP thymocytes was increased significantly. In contrast, the ratios of CD4-SP and CD8-SP thymocytes decreased significantly in the irradiated mice with BMT compared with those in total thymocytes from the control mice (***Fig. 4C***, right panel, ***Table 3***). These results suggest that radiation impairs the maturation of donor-derived DP thymocytes in recipient thymus.

**Table 2:** Role of bone marrow transplantation (BMT) in the regeneration of thymocytes of different stages in irradiated mice

Cell type	Constituent ratio (%)			Cellularity (×10^6^)		
	Untreated	XR	XR+BMT	Untreated	XR	XR+BMT
CD4^–^CD8^–^	8.21±2.08	14.17±1.88*	6.96±2.43^#^	07.07±1.55	1.67±0.85**	6.48±1.91^#^
CD4^+^CD8^+^	82.63±2.10	49.87±1.91***	83.03±0.72^###^	71.95±7.97	5.70±2.08***	78.91±9.79^###^
CD4^+^CD8^–^	06.65±0.69	20.53±1.46***	07.77±1.95^###^	05.75±0.07	2.31±0.69**	06.45±2.48^#^
CD4^–^CD8^+^	02.50±0.50	15.40±0.60***	02.25±0.16^###^	02.19±0.59	1.76±0.66	02.65±0.38

*P < 0.05, **P < 0.01, ***P < 0.001 compared with the untreated group; ^#^*P* < 0.05, ^###^*P* < 0.001 compared with the irradiated (XR) group.

(mean±SD, *n* = 5)

To determine whether the impaired maturation of donor-derived DP thymocytes in recipient thymus is associated with the down-regulation of Notch signaling, thymic tissue sections were stained immunohistochemically for Jagged1, a Notch ligand. The results showed that Jagged1 expression was detected in the cortical epithelia and was reduced in the irradiated thymus with BMT compared to untreated thymus (***Fig. 4D***). This result suggests that reduced expression of Jagged1 may play a significant role in radiation-impaired maturation of donor-derived DP thymocytes in recipient thymus.

**Table 3:** Maturation of donor cells in the thymus of irradiated mice

Cell type	Constituent ratio (%)	
	Untreated	GFP^+^ (XR+BMT)
CD4^–^CD8^–^	08.21±2.08	05.92±0.56
CD4^+^CD8^+^	82.63±2.10	89.20±1.10**
CD4^+^CD8^–^	06.65±0.69	03.58±0.18**
CD4^–^CD8^+^	02.50±0.49	0 1.54±0.27*

*P < 0.05, **P < 0.01 compared with the control group. BMT: bone marrow transplantation; XR: X-ray irradiation.

(mean±SD, *n* = 5)

## DISCUSSION

Previous studies have reported that radiation induces acute thymic atrophy and reduces the total number of thymocytes[18],[19], especially DP thymocytes[4]. Our results are consistent with these findings. In addition to DP thymocytes, other thymocytes of different stages also cannot escape from radiation-induced cell death, especially DN thymocytes, which suffered a 76.38% loss. Previous studies have mainly focused on the great sensitivity of DP thymocytes to radiation[4],[20]. However, what contributes to the different sensitivity of thymocytes of different stages to radiation-induced cell death is unclear. The viewpoint that proliferative cancer cells are sensitive to radiation damage has been widely accepted in cancer therapy. Therefore, we raised the questions whether different proliferation exists among thymocytes of different stages and whether the degree of radiosensitivity of thymocytes of different stages correlates with their proliferation rates. Our results demonstrated the following facts: 1) Different proliferation exists in thymocytes of different stages; 2) There was a significant correlation between the percentage of thymocytes maintained at G_2_/M phase and the percentage of radiation-induced cell death in the thymus. These results provide strong evidence that the degree of radiosensitivity of thymocytes depends on their proliferative states and would shed some light on elucidating the mechanism of radiation-induced damage on thymocytes.

In the present study, irradiated mice were transplanted with donor BM cells from GFP transgenic mice. At 4 weeks after transplantation, the size and cellularity of the thymus and a totally rescued percentage of thymocytes of different stages were detected. We found that the total number of GFP-negative thymocytes and percentages of thymocytes of different stages in GFP-negative thymocytes are comparable between the irradiated mice and the irradiate mice with BMT, suggesting that reconstruction of the thymus from the irradiated mice with BMT is completely due to donor-derived BM cells. However, our results demonstrated that the percentage of GFP-positive DP thymocytes increased significantly, whereas the percentages of GFP-positive CD4-SP and CD8-SP thymocytes decreased significantly in the irradiated mice with BMT compared with untreated mice. These results indicated that the maturation of donor-cells in the thymus of irradiated mice was not completed, with a dysfunctional transition from DP thymocytes to SP thymocytes. The maturation of T-cell precursors is a non-cell autonomous process, and the interactions of cells with the thymic microenvironment are required for T-cell development[21]–[23]. The notch signaling pathway is critical to the process of thymocytes maturation, including positive[14],[15] and negative[16] selection of DP thymocytes. Notch activation requires binding of Notch ligand, such as Jagged1, which is expressed by a cortical epithelial subset[17]. Our results demonstrated that Jagged1 expression was significantly down-regulated in thymic cortical epithelia in the irradiated mice with BMT, suggesting that the transition dysfunction of donor-derived DP thymocytes is possibly related to the damaged thymic microenvironment mediated by Notch signaling.

Post-BMT immunodeficiency causes significant morbidity and mortality in BMT recipients[24]–[26]. Although neutropenia-related infections predominate in the immediate post-transplantation period, infections after myeloid engraftment are generally due to bacterial, fungal, and viral pathogens that are controlled by the adaptive immune system[27]. Therefore, it is very important to promote the generation of functional SP T lymphocytes in the functional recovery of recipient immune system. In our study, despite the engraftment of donor BM cells into recipient thymus and the recovery of partial thymocytes at different stages, the generation of functional T lymphocytes in the recipient was delayed, possibly owing to the damage of the thymic microenvironment. These results suggest that a graft containing a mixture of thymic epithelia and BM cells would be a good choice for transplantation. This graft containing mixture would be beneficial to the functional reconstruction (including recovery of thymocytes and the thymic microenvironment) of the thymus in irradiated recipient.

In summary, our results demonstrated that the degree of radiosensitivity of thymocytes depends on their proliferative states and radiation impairs the maturation of donor-derived CD4^+^CD8^+^ thymocytes cells in recipient thymus.
