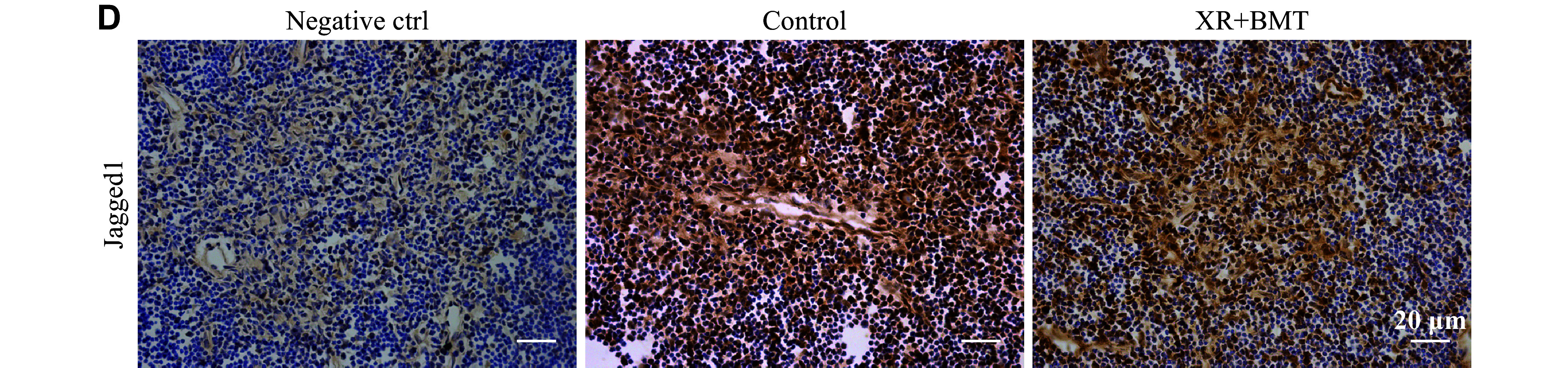# Author Correction: X-ray irradiation selectively kills thymocytes of different stages and impairs the maturation of donor-derived CD4^+^CD8^+^ thymocytes in recipient thymus

**DOI:** 10.7555/JBR.38.20249001

**Published:** 2025-03

**Authors:** Jinbo Li, Hongquan Cai, Jianliang Jin, Qian Wang, Dengshun Miao

**Affiliations:** 1 The Research Center for Bone and Stem Cells, Department of Human Anatomy, Nanjing Medical University, Nanjing, Jiangsu 210029, China; 2 Department of Preventive Medicine, School of Public Health, Nanjing Medical University, Nanjing, Jiangsu 210029, China

Correction to: Journal of Biomedical Researchhttps://doi.org/10.7555/JBR.26.20120003, published on June 8, 2012.

We sincerely apologize for the misuse of images in ***Fig. 4*** of our article. Specifically, the image in ***Fig. 4D***, depicting immunohistochemistry staining for Jagged1 expression, was partially incorrect because of our oversight. Upon reviewing the laboratory records, we have successfully located the original images from 12 years ago. We are now providing the corrected version of ***Fig. 4D*** below. This correction does not alter the conclusions as originally reported.

**Figure 4D:**